# The efficacy of traditional chinese medicine combined with hyperthermic intraperitoneal chemotherapy for malignant ascites: A systematic review and meta-analysis

**DOI:** 10.3389/fphar.2022.938472

**Published:** 2022-08-29

**Authors:** Zhixian Lin, Jiangfeng Chen, Yunxia Liu

**Affiliations:** ^1^ Department of Oncology, Hangzhou Third People’s Hospital, Hangzhou, Zhejiang, China; ^2^ Department of Integrated Traditional Chinese & Western Medicine Oncology, Hangzhou Cancer Hospital, Hangzhou, Zhejiang, China

**Keywords:** hyperthermic intraperitoneal chemotherapy, malignant ascites, traditional Chinese medicine, efficacy, meta-analysis

## Abstract

**Objective:** Malignant ascites (MA) is a common complication of terminal cancer, which seriously affects the life quality and prognosis of patients. Both hyperthermic intraperitoneal chemotherapy (HIPEC) and traditional Chinese medicine (TCM) preparations have achieved significant efficacy in the treatment of MA. The treatment strategy of TCM combined with HIPEC has been gradually promoted and applied in China. The purpose of this systematic review and meta-analysis was to assess the efficacy of TCM combined with HIPEC in the treatment of MA.

**Methods:** Randomized controlled trials (RCTs) of TCM combined with HIPEC for MA were searched from seven electronic databases. Two researchers used the Cochrane Collaboration’s tool to assess the risk of bias. Excel 2019 was used to establish a database for information extraction, RevMan 5.4 software was used to analyze the included test data, and STATA v16.0 was used to conduct Egger’s test to further detect publication bias.

**Results:** A total of 19 studies involving 1,504 patients were included in this meta-analysis. The results showed that compared with the single use of HIPEC, TCM combined with HIPEC could significantly improve the clinical efficacy (RR = 1.51, 95% CI [1.40, 1.63], *p* < 0.00001) and karnofsky performance status (KPS) score (MD = 8.16, 95% CI [6.46, 9.85], *p* < 0.00001), reduce the ascites volume (MD = −156.98, 95% CI [−213.71, −100.25], *p* < 0.00001). However, there was no statistical significance in reducing abdominal circumference between TCM combined with HIPEC and HIPEC alone (MD = −1.8, 95% CI [−4.57, −0.97], *p* = 0.2).

**Conclusion:** This study found that TCM combined with HIPEC had a beneficial therapeutic effect on MA. However, more standard, double-blind, multicenter RCTs are needed to further confirm the efficacy of TCM combined with HIPEC in the treatment of MA.

**Systematic Review Registration:** https://www.crd.york.ac.uk/, identifier CRD42022319993.

## Introduction

Malignant ascites (MA) is a common complication of peritoneal disseminated tumors such as ovarian cancer, breast cancer, intestinal cancer, gastric cancer and pancreatic cancer (Becker et al., 2006). Modern medicine believes that its mechanism is mostly related to the damage of abdominal wall serosa and secretion of certain mediators by metastatic cancer cells in the abdominal wall, resulting in increased peritoneal vascular permeability, excessive fluid production, and hypoproteinemia, which further leads to hydrodynamic imbalance, portal vein obstruction, subdiaphragmatic lymphatic vessel and vein return disorders (Zhan et al., 2016; Ito and Hanafusa, 2017). If no effective intervention measures are taken for patients with MA, symptoms such as abdominal distention, abdominal pain, nausea, vomiting, and even dyspnea may occur, seriously affecting the life quality and prognosis of patients (Maeda et al., 2015; Hodge and Badgwell, 2019).

Commonly used methods for the clinical treatment of MA include abdominal paracentesis drainage, diuretics, chemotherapy, immunotherapy, targeted therapy, etc. These treatment methods often have disadvantages such as side effects, high price and drug resistance, which limit the overall therapeutic effect. Hyperthermic intraperitoneal chemotherapy (HIPEC) is an emerging treatment method for peritoneal disseminated tumors in recent years, which has a wide range of indications, simple operation and remarkable efficacy, and has achieved certain efficacy in the control of MA (Robella et al., 2019). However, in actual clinical practice, due to the differences of individual efficacy, single use of HIPEC cannot completely and effectively control the condition of each patient. In clinical practice, appropriate and effective treatment plans need to be formulated according to the specific situation of patients.

Traditional Chinese medicine (TCM) is a medical system with unique theoretical style and characteristics of diagnosis and treatment formed gradually in long-term medical practice. Studies have shown that TCM treatment can effectively curb the generation of MA, alleviate the toxicity of chemotherapy, relieve the clinical symptoms of patients, improve the immune function and quality of life of patients, and prolong the survival period, and TCM treatment has few side effects and high safety (Kong, 2021). TCM is considered to be used in combination with HIPEC in patients with MA, which can improve the efficacy of drugs and reduce the side effects of drugs, and is widely used in the treatment of MA (Wang, 2020). Therefore, it is of great significance to further evaluate the clinical value of HIPEC combined with TCM on the basis of evidence-based medicine. This paper was based on a systematic review of randomized controlled trial (RCTS) of TCM including oral and external administration combined with HIPEC for MA. The purpose is to obtain evidence of the efficacy and safety of TCM adjuvant treatment of MA, and to provide evidence-based medical basis for its treatment.

## Methods

### Study registration

This systematic review and meta-analysis followed the PRISMA guidelines and were registered in the PROSPERO database (https://www.crd.york.ac.uk/). The registration number is CRD42022319993.

### Search strategy

We searched PubMed, Cochrane Library, Embase, China National Knowledge Infrastructure (CNKI), Wanfang Database, China Science and Technology Journal Database (VIP) and Sinomed. The search time was from establishment to 20 March 2022. The following search terms were used: “traditional Chinese medicine,” “herbs,” “ascites,” “peritoneal effusion,” “malignant ascites,” “malignant peritoneal effusion,” “hyperthermic perfusion,” “hyperthermic intraperitoneal chemotherapy,” “peritoneal hyperthermic perfusion chemotherapy.” Details of the search strategies were available in Supplementary Material. In addition, we searched conference literature and clinical registry data for relevant trials that might have been missing in web searches. This study was conducted independently by two researchers (JC and ZL).

### Inclusion and exclusion criteria

Inclusion criteria were as follows: 1) Study design: All included studies were RCTs. 2) Participants: Malignant tumors were diagnosed by histopathology and/or cytology. At the same time, ascites was confirmed by imaging examination (B-ultrasound and/or computed tomography) and cancer cells were found in ascites cytology. 3) Interventions: The control group received HIPEC, in combination with or without intravenous chemotherapy, with no restrictions on the drugs for HIPEC, dosage or course of treatment. On the basis of the control group, the trial group was given oral or external TCM treatment, and there were not any restrictions on the prescription composition, dosage and course of treatment of TCM. 4) Outcomes: The main outcome was clinical efficacy. The efficacy was evaluated according to the malignant tumor related curative effect standards formulated by WHO (Xu, 2005). Complete remission (CR) is defined as complete absorption of ascites with a duration >4 weeks. Partial remission (PR) is the reduction of ascites ≥50% and the duration >4 weeks. Stable disease (SD) is a decrease in ascites <50% or increase in ascites <25%. Progression disease (PD) is a significant increase in ascites ≥25%. Clinical efficacy = CR + PR. The secondary outcomes were the comparison of ascites volume, abdominal circumference, and karnofsky performance status (KPS) score after treatment. The included studies reported at least one of the above outcomes.

Exclusion criteria were as follows: 1) The full text cannot be obtained through electronic retrieval, manual retrieval and author’s email. 2) Studies with the following patients: Patients with severe heart, liver, kidney and other organic diseases; patients complicated with blood system, immune system and serious infectious diseases; patients with severe peritoneal adhesion; ascites caused by other diseases; patients with contraindications to chemotherapy. 3) Drugs for HIPEC included non-chemotherapy drugs in the studies. 4) The intervention measures in the trial group included TCM injection. 5) Studies that lacked outcome data or cannot be analyzed.

### Study selection and data extraction

Two researchers (JC and ZL) carried out the study selection and data extraction independently, and the third researcher (YL) resolved any disagreement. Using Excel 2019 establishing database of information extraction, we extracted the following information from the included studies: general information (the first author, year of publication), participants characteristics (age, gender, sample size, type of cancer), interventions (drugs for HIPEC, ways of using TCM, herbs in prescriptions, course of treatment), and outcomes.

### Risk of bias assessment

Two researchers (JC and ZL) independently assessed the risk of bias for included studies using the Cochrane Collaboration’s risk of bias tool (Higgins et al., 2011). The risk of bias assessment tool includes the following assessments: random sequence generation (selection bias), allocation concealment (selection bias), blinding of participants and personnel (performance bias), blinding of outcome assessment (detection bias), incomplete outcome data (attrition bias), selective reporting (reporting bias), and other bias. After the risk of bias assessment, each item can be classified as “high risk of bias,” “risk of unilateral bias” or “low risk of bias.” If there is a difference in the assessment results of two researchers, it can be resolved by consultation with the third researcher.

### Data analysis

RevMan (Review Manager 5.4) statistical software provided by the Cochrane Collaboration was used for data analysis (Cochrane, 2012). Dichotomous data were expressed as risk ratio (RR) with 95% confidence interval (CI). Continuous data were expressed as mean difference (MD) with 95% CI. Chi-square and I-Square (I^2^) indexes were used for heterogeneity test. When *p* ≥ 0.05 and I^2^ ≤ 50%, the fixed-effect model was selected. Otherwise, the random-effect model was selected. We conducted subgroup analysis according to whether it was combined with intravenous chemotherapy, different ways of using TCM, different drugs for HIPEC, and different course of treatment to further verify the efficacy of TCM combined with HIPEC in the treatment of MA. *p* ≤ 0.05 was considered statistically significant, and all tests were two-sided tests. In addition, for a single outcome, if the number of studies analyzed exceeds 10, a funnel plot will be drawn to assess the existence of publication bias (Pérez and Rodríguez, 2006), and STATA v16.0 was used to conduct Egger’s test to further detect publication bias (Egger et al., 1997). Sensitivity analysis was performed by removing individual studies to assess the stability of the results. In addition, Excel software was used to analyze the frequency and medicinal properties of herbs. Pharmacopoeia of the People’s Republic of China (The Chinese pharmacopoeia, the first part, 2015) was the standard for the names and efficacy classifications of herbs in prescriptions, supplemented by the Traditional Chinese Pharmacology and Chinese Materia Medica (Qiu, 2006; Gao, 2010).

## Results

### Study selection

**FIGURE 1:**
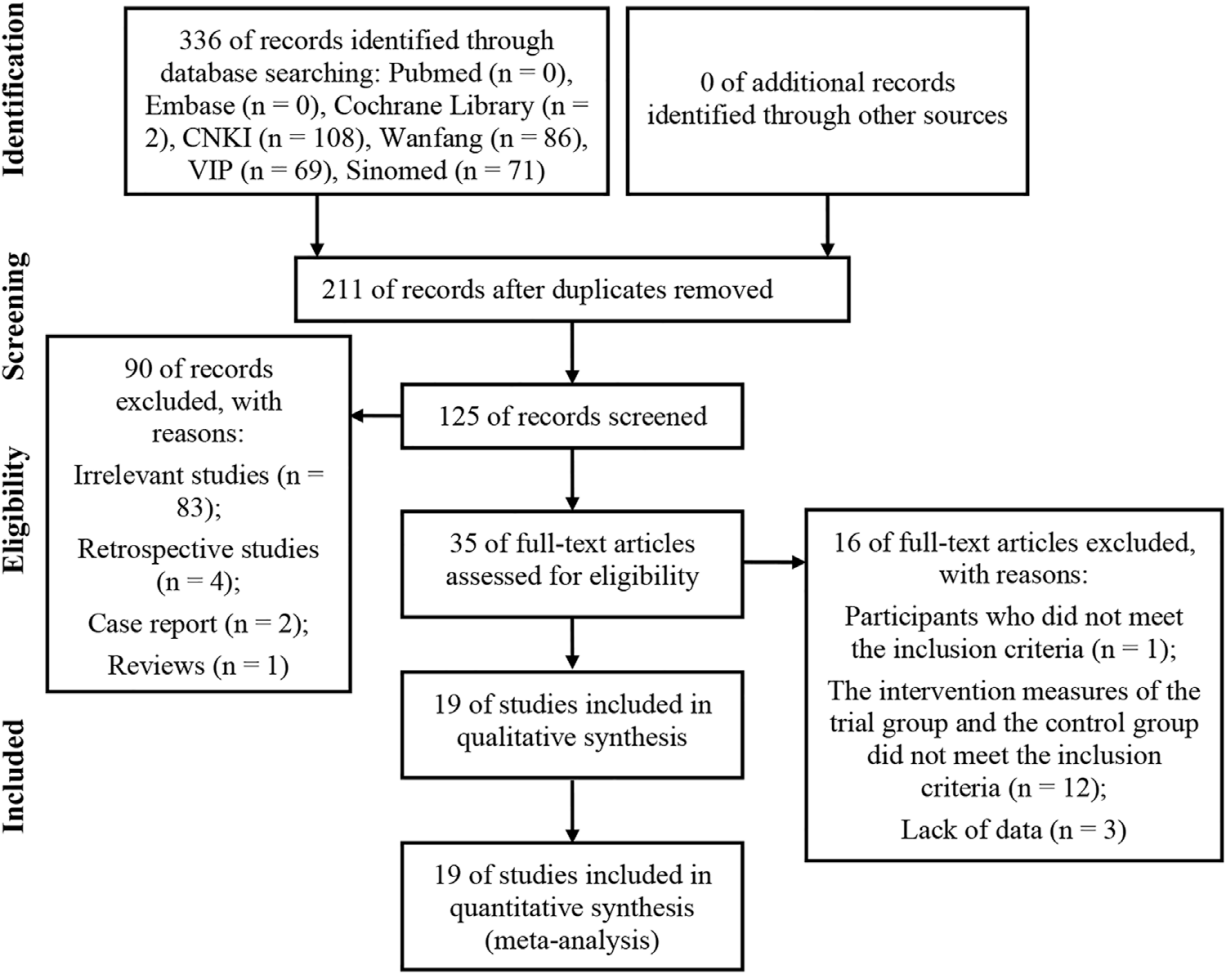
Flow chart of study identification and selection.

We retrieved 336 potentially relevant articles from seven electronic databases (PubMed, Cochrane Library, Embase, CNKI, Wanfang database, VIP and Sinomed). After we used EndNote software to delete duplicate articles, we retained 125 studies for further confirmation. Then, by reading titles and abstracts, 90 articles (83 unrelated studies, four retrospective studies, 2 case reports and one review) that did not meet the criteria for full text review were deleted. After reading the full text of the remaining 35 articles, 16 articles were further removed (1 participant did not meet the inclusion criteria, 12 interventions in the control and trial groups did not meet the inclusion criteria, and three lacked outcome data). Finally, we included 19 eligible studies for comprehensive analysis. The screening process was shown in Figure 1.

### Characteristics of included studies

A total of 19 RCTs were included in this study, with a total of 1,504 patients, including 762 patients in the trial group and 742 patients in the control group. All studies were conducted in China between 2010 and 2021. Seven of the studies (Wen et al., 2015; Dai et al., 2016; Chen and Hua, 2017; Gao and Zhang, 2017; Zhang et al., 2018; Shao et al., 2019; Rui and Peng, 2021) included only ovarian cancer patients, one study (Zhang and Li, 2018) only included gastric cancer patients, nine studies (Cui and Wu, 2010; Wu et al., 2016; Pan et al., 2017; Zhang et al., 2017; Jiang and Hu, 2019; Cai and Zhu, 2020; Li et al., 2020; Mei et al., 2020; Li, 2021) included multiple tumor types, two studies (Li, 2020; Zhang et al., 2020) did not report the tumor type. The control group in five studies (Wu et al., 2016; Pan et al., 2017; Zhang et al., 2018; Shao et al., 2019; Rui and Peng, 2021) were treated with HIPEC combined with intravenous chemotherapy, and the trial group were treated with TCM on this basis, and the control group in remaining 14 studies (Cui and Wu, 2010; Wen et al., 2015; Dai et al., 2016; Chen and Hua, 2017; Gao and Zhang, 2017; Zhang et al., 2017; Zhang and Li, 2018; Jiang and Hu, 2019; Cai and Zhu, 2020; Li, 2020; Li et al., 2020; Mei et al., 2020; Zhang et al., 2020; Li, 2021) only received HIPEC, and the trial group added TCM on this basis. One study (Dai et al., 2016) used paclitaxel single agent for HIPEC, one study (Zhang and Li, 2018) used 5-fluorouracil and cisplatin for HIPEC, and the remaining 17 studies (Cui and Wu, 2010; Wen et al., 2015; Wu et al., 2016; Chen and Hua, 2017; Gao and Zhang, 2017; Pan et al., 2017; Zhang et al., 2017; Zhang et al., 2018; Jiang and Hu, 2019; Shao et al., 2019; Cai and Zhu, 2020; Li, 2020; Li et al., 2020; Mei et al., 2020; Zhang et al., 2020; Li, 2021; Rui and Peng, 2021) used cisplatin single agent for HIPEC. The trial group in 15 studies (Cui and Wu, 2010; Dai et al., 2016; Wu et al., 2016; Chen and Hua, 2017; Gao and Zhang, 2017; Pan et al., 2017; Zhang et al., 2018; Jiang and Hu, 2019; Shao et al., 2019; Cai and Zhu, 2020; Li, 2020; Mei et al., 2020; Zhang et al., 2020; Li, 2021; Rui and Peng, 2021) was treated with oral administration of TCM, the trial group in four studies (Wen et al., 2015; Zhang et al., 2017; Zhang and Li, 2018; Li et al., 2020) were treated with external application of TCM. Table 1 summarized the patient characteristics of the included studies, including age, gender, sample size, interventions, the course of treatment, outcomes, etc.

**TABLE 1:** Baseline characteristics of included studies.

First author (year)	Age/years	Gender		Sample size		Types of cancer	Intervention measures			Outcomes
		Male	Female	Trial group	Control group		Trial group	Control group	Course of treatment	
Rui and Peng (2021)	48–72	NR	NR	47	46	Ovarian Cancer	Intravenous chemotherapy with paclitaxel + Hyperthermic intraperitoneal chemotherapy with cisplatin + Oral traditional Chinese medicine	Intravenous chemotherapy with paclitaxel + Hyperthermic intraperitoneal chemotherapy with cisplatin	The interval of chemotherapy was 3–4 weeks, and the course of treatment is 4–6 cycles in total	Clinical Efficacy, KPS
Pan et al. (2017)	30–70	43	35	39	39	Liver Cancer, Ovarian Cancer, Intestinal Cancer, Gastric Cancer, Others	Intravenous chemotherapy + Hyperthermic intraperitoneal chemotherapy with cisplatin + Oral traditional Chinese medicine	Intravenous chemotherapy + Hyperthermic intraperitoneal chemotherapy with cisplatin	The treatment lasted for 7–10 days for 12 cycles	Clinical Efficacy
Wu et al. (2016)	22–80	42	57	50	49	Breast Cancer, Ovarian Cancer, Gastric Cancer, Colon Cancer, Rectal Cancer, Endometrial Cancer	Intravenous chemotherapy + Hyperthermic intraperitoneal chemotherapy with cisplatin + Oral traditional Chinese medicine	Intravenous chemotherapy + Hyperthermic intraperitoneal chemotherapy with cisplatin	12 weeks	Clinical Efficacy, KPS
Zhang et al. (2018)	NA	0	85	44	41	Ovarian Cancer	Intravenous chemotherapy with paclitaxel + Hyperthermic intraperitoneal chemotherapy with cisplatin + Oral traditional Chinese medicine	Intravenous chemotherapy with paclitaxel + Hyperthermic intraperitoneal chemotherapy with cisplatin	8 weeks	Clinical Efficacy, KPS, Ascites Volume
Shao et al. (2019)	53–74	0	100	50	50	Ovarian Cancer	Intravenous chemotherapy with paclitaxel + Hyperthermic intraperitoneal chemotherapy with cisplatin + Oral traditional Chinese medicine	Intravenous chemotherapy with paclitaxel + Hyperthermic intraperitoneal chemotherapy with cisplatin	8 weeks	Clinical Efficacy, Ascites Volume
Li (2020)	36–73	33	27	30	30	NR	Hyperthermic intraperitoneal chemotherapy with cisplatin + Oral traditional Chinese medicine	Hyperthermic intraperitoneal chemotherapy with cisplatin	2 weeks	Clinical Efficacy, KPS
Cui and Wu (2010)	35–76	47	39	50	36	Gastric Cancer, Colorectal Cancer, Ovarian Cancer, Breast Cancer	Hyperthermic intraperitoneal chemotherapy with cisplatin + Oral traditional Chinese medicine	Hyperthermic intraperitoneal chemotherapy with cisplatin	Until the ascites is controlled or local therapy is stopped	Clinical Efficacy
Cai and Zhu (2020)	54–79	30	30	30	30	Gastric Cancer, Intestinal Cancer, Pancreatic Cancer	Hyperthermic intraperitoneal chemotherapy with cisplatin + Oral traditional Chinese medicine	Hyperthermic intraperitoneal chemotherapy with cisplatin	2 weeks	Clinical Efficacy, Abdominal Circumference
Li (2021)	38–70	22	35	29	28	Gastric Cancer, Liver Cancer, Colorectal Cancer, Pancreatic Cancer, Ovarian Cancer	Hyperthermic intraperitoneal chemotherapy with cisplatin + Oral traditional Chinese medicine	Hyperthermic intraperitoneal chemotherapy with cisplatin	4 weeks	Abdominal Circumference, KPS
Jiang and Hu (2019)	20–75	52	27	40	40	Gastric Cancer, Ovarian Cancer, Colorectal Cancer, Pancreatic Cancer, Liver Cancer	Hyperthermic intraperitoneal chemotherapy with cisplatin + Oral traditional Chinese medicine	Hyperthermic intraperitoneal chemotherapy with cisplatin	4 weeks	Clinical Efficacy, KPS
Chen and Hua (2017)	54–74	0	100	50	50	Ovarian Cancer	Hyperthermic intraperitoneal chemotherapy with cisplatin + Oral traditional Chinese medicine	Hyperthermic intraperitoneal chemotherapy with cisplatin	8 weeks	Clinical Efficacy, Ascites Volume, KPS
Mei et al. (2020)	40–62	0	60	30	30	Endometrial Cancer, Ovarian Cancer, Cervical Cancer	Hyperthermic intraperitoneal chemotherapy with cisplatin + Oral traditional Chinese medicine	Hyperthermic intraperitoneal chemotherapy with cisplatin	4 weeks	Clinical Efficacy, KPS
Dai et al. (2016)	56–68	0	60	30	30	Ovarian Cancer	Hyperthermic intraperitoneal chemotherapy with paclitaxel + Oral traditional Chinese medicine	Hyperthermic intraperitoneal chemotherapy with paclitaxel	9–12 weeks	Clinical Efficacy, KPS
Zhang et al. (2020)	46–70	50	30	40	40	NR	Hyperthermic intraperitoneal chemotherapy with cisplatin + Oral traditional Chinese medicine	Hyperthermic intraperitoneal chemotherapy with cisplatin	4 weeks	Clinical Efficacy
Gao and Zhang (2017)	55–70	0	100	50	50	Ovarian Cancer	Hyperthermic intraperitoneal chemotherapy with cisplatin + Oral traditional Chinese medicine	Hyperthermic intraperitoneal chemotherapy with cisplatin	6–8 weeks	Clinical Efficacy, KPS
Zhang et al. (2017)	28–70	31	45	38	38	Gastric Cancer, Ovarian Cancer, Intestinal Cancer, Breast Cancer, Endometrial Cancer, Liver Cancer, Pancreatic Cancer, Nasal Rhabdomyosarcoma, Esophagus Cancer, Lymphoma, Lung Cancer	Hyperthermic intraperitoneal chemotherapy with cisplatin + External traditional Chinese medicine	Hyperthermic intraperitoneal chemotherapy with cisplatin	2 weeks	Clinical Efficacy, Abdominal Circumference, KPS
Zhang et al. (2018)	35–76	65	41	53	53	Gastric Cancer	Hyperthermic intraperitoneal chemotherapy with cisplatin and 5-fluorouracil + External traditional Chinese medicine	Hyperthermic intraperitoneal chemotherapy with cisplatin and 5-fluorouracil	4 weeks	Clinical Efficacy
Li et al. (2020)	44–81	34	40	37	37	Gastric Cancer, Intestinal Cancer, Lung Cancer, Ovarian Cancer, Liver Cancer, Esophagus Cancer, Pancreatic Cancer, Gallbladder Cancer, Abdominal Myxoadenoma	Hyperthermic intraperitoneal chemotherapy with cisplatin + External traditional Chinese medicine	Hyperthermic intraperitoneal chemotherapy with cisplatin	4 weeks	Clinical Efficacy, Abdominal Circumference, KPS
Wen et al. (2015)	30–82	0	50	25	25	Ovarian Cancer	Hyperthermic intraperitoneal chemotherapy with cisplatin + External traditional Chinese medicine	Hyperthermic intraperitoneal chemotherapy with cisplatin	3 weeks	Clinical Efficacy, KPS

NR: not reported; KPS: karnofsky performance status.

### Risk of bias assessment

**FIGURE 2:**
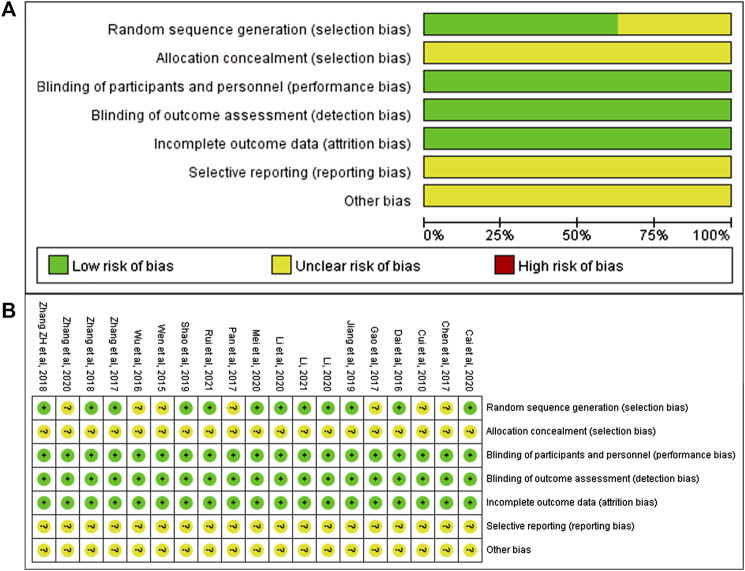
Risk of bias of included studies. **(A)** Risk of bias graph; **(B)** Risk of bias summary.

We used the Cochrane Collaboration’s risk of bias tool to assess the quality of studies. 1) Selection bias (Random sequence Generation): 11 studies (Dai et al., 2016; Zhang et al., 2017; Zhang et al., 2018; Zhang and Li, 2018; Jiang and Hu, 2019; Shao et al., 2019; Li, 2020; Li et al., 2020; Mei et al., 2020; Li, 2021; Rui and Peng, 2021) used a random table for random allocation, and one study (Cai and Zhu, 2020) used draw method for random allocation, and the risk of selection bias was considered “low.” Seven studies (Cui and Wu, 2010; Wen et al., 2015; Wu et al., 2016; Chen and Hua, 2017; Gao and Zhang, 2017; Pan et al., 2017; Zhang et al., 2020) claimed that randomization was used, but did not report details of how randomization was done, and the risk of selection bias was considered “unclear.” 2) Selection bias (allocation concealment): None of the studies reported allocation concealment, and the risk of selection bias was considered “unclear.” 3) Performance bias and detection bias: None of the studies showed whether blind method was used. However, considering that these studies used objective outcome indicators, the results were not interfered by researchers and participants. Therefore, the risk of performance bias and detection bias were considered “low.” 4) Attrition bias: There was no missing data in all included studies, so the risk of attrition bias was considered “low.” 5) Reporting bias and other bias: None of the studies had enough information to assess whether there was a risk of selective reporting and other bias, and therefore they were identified as “unclear risks.” All risk of bias assessment data was shown in Figure 2.

### Frequency statistics of traditional chinese medicine

The frequency of each herb in the prescription for MA was ranked from high to low. The top three were atractylodes macrocephala Koidz, poria cocos (Schw.) Wolf, astragalus membranaceus (Fisch.) Bunge, as shown in the Table 2. We made statistics on the medicinal properties of herbs commonly used in MA. The top three were qi-invigorating herbs, herbs for inducing diuresis and excreting, heat-clearing herbs, as shown in the Table 3.

**TABLE 2:** Frequency of TCM.

TCM	Frequency
Atractylodes macrocephala Koidz	14
Poria cocos (Schw.)Wolf	11
*Astragalus* membranaceus (Fisch.) Bunge	8
Aconitum carmichaelii Debx	7
Curcuma phaeocaulis Valeton	6
Hedyotis diffusa Willd	6
*Glycyrrhiza* uralensis Fisch	6
*Cynanchum* otophyllum Schneid	6
Zingiber officinale Roscoe	5
Polyporus	5
Alisma orientalis (Sam.)Juzep	5
Cinnamomum cassia Presl	4
Coix lacryma-jobi L.var.mayuen (Roman.) Stapf	4
E. brevicornum Maxim	4
Areca Peel	4
*Euphorbia* kansui T. N. Liou ex S. B. Ho	3
Salvia miltiorrhiza Bge	3

**TABLE 3:** Frequency of medicinal properties of TCM.

Medicinal properties of herbs	Frequency	Frequency rate (%)
Qi-invigorating herbs	42	21.762
Herbs for inducing diuresis and excreting	28	14.508
Heat-clearing herbs	22	11.399
Blood-activating and stasis-eliminating herbs	20	10.363
Interior-warming herbs	10	5.181
Blood-tonifying herbs	9	4.663
Diaphoretic herbs	9	4.663
Qi-regulating herbs	9	4.663
Purging herbs	9	4.663
Yang-reinforcing herbs	8	4.145
Yin-reinforcing herbs	6	3.109
Hemostatic herbs	3	1.554
Agent that relieves toxicity, kills insects and stops itching	3	1.554
Digestive herbs	3	1.554
Herbs for facilitating expectoration, suppressing cough and relieving dyspnea	3	1.554
Herbs for calming liver to stop endogenous wind	3	1.554
Damp dispersing herbs	2	1.036
Astringent therapy herbs	2	1.036
Wind-damp dispelling herbs	2	1.036

## Outcomes measures

### Clinical efficacy

**FIGURE 3:**
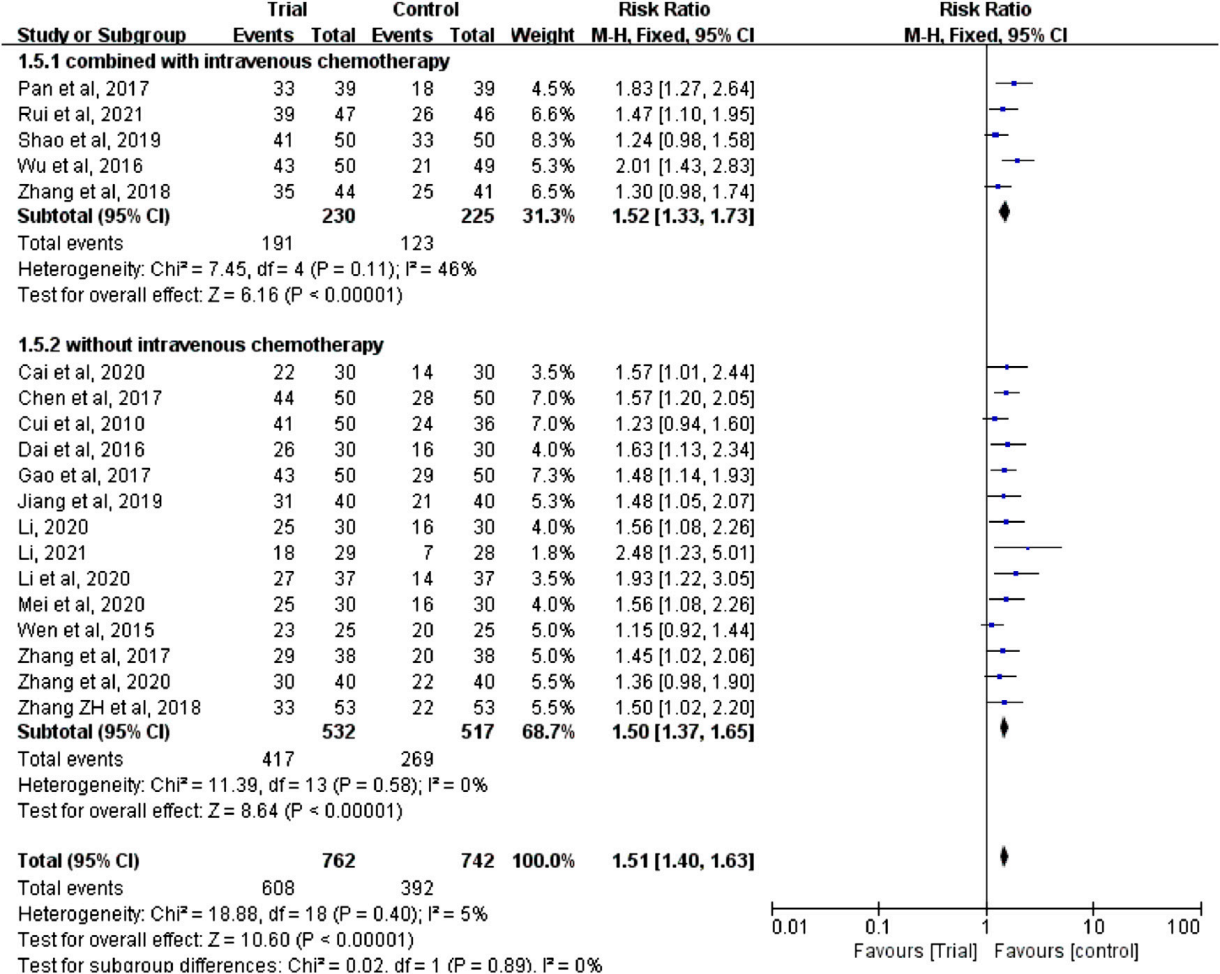
Subgroup analysis of clinical efficacy according to whether combined with intravenous chemotherapy.

Nineteen studies (Cui and Wu, 2010; Wen et al., 2015; Dai et al., 2016; Wu et al., 2016; Chen and Hua, 2017; Gao and Zhang, 2017; Pan et al., 2017; Zhang et al., 2017; Zhang et al., 2018; Zhang and Li, 2018; Jiang and Hu, 2019; Shao et al., 2019; Cai and Zhu, 2020; Li, 2020; Li et al., 2020; Mei et al., 2020; Zhang et al., 2020; Li, 2021; Rui and Peng, 2021) reported clinical efficacy with a total of 1,504 patients, including 762 patients in the trial group and 742 patients in the control group. Due to the low heterogeneity of the data (I^2^ = 5%, *p* = 0.4), a fixed-effect model was used. The results showed that the clinical efficacy of TCM combined with HIPEC (608/762) was significantly better than that of the single HIPEC (392/742) (RR = 1.51, 95% CI [1.40, 1.63], *p* < 0.00001, Figure 3).

**FIGURE 4:**
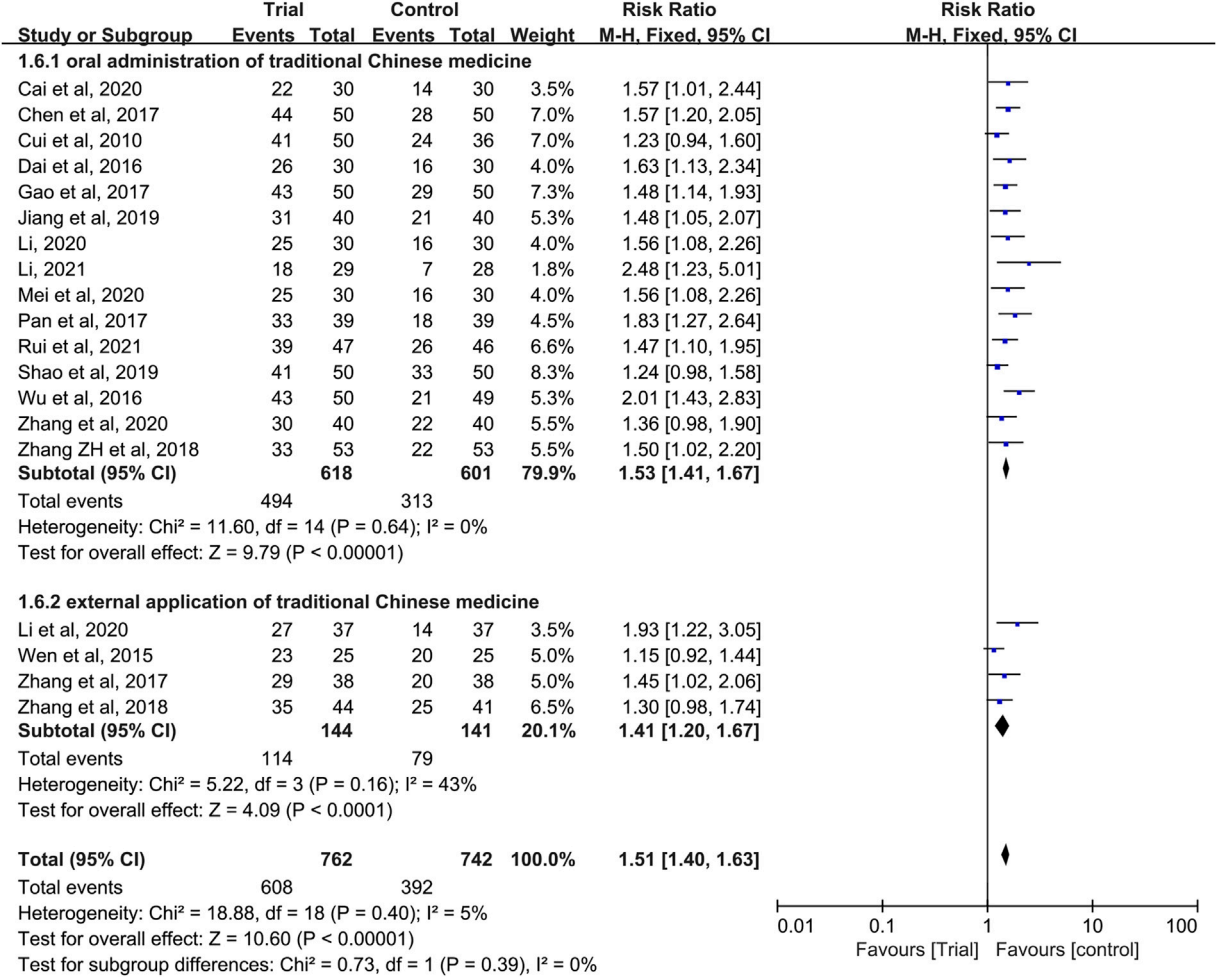
Subgroup analysis of clinical efficacy according to different ways of using traditional Chinese medicine.

**FIGURE 5:**
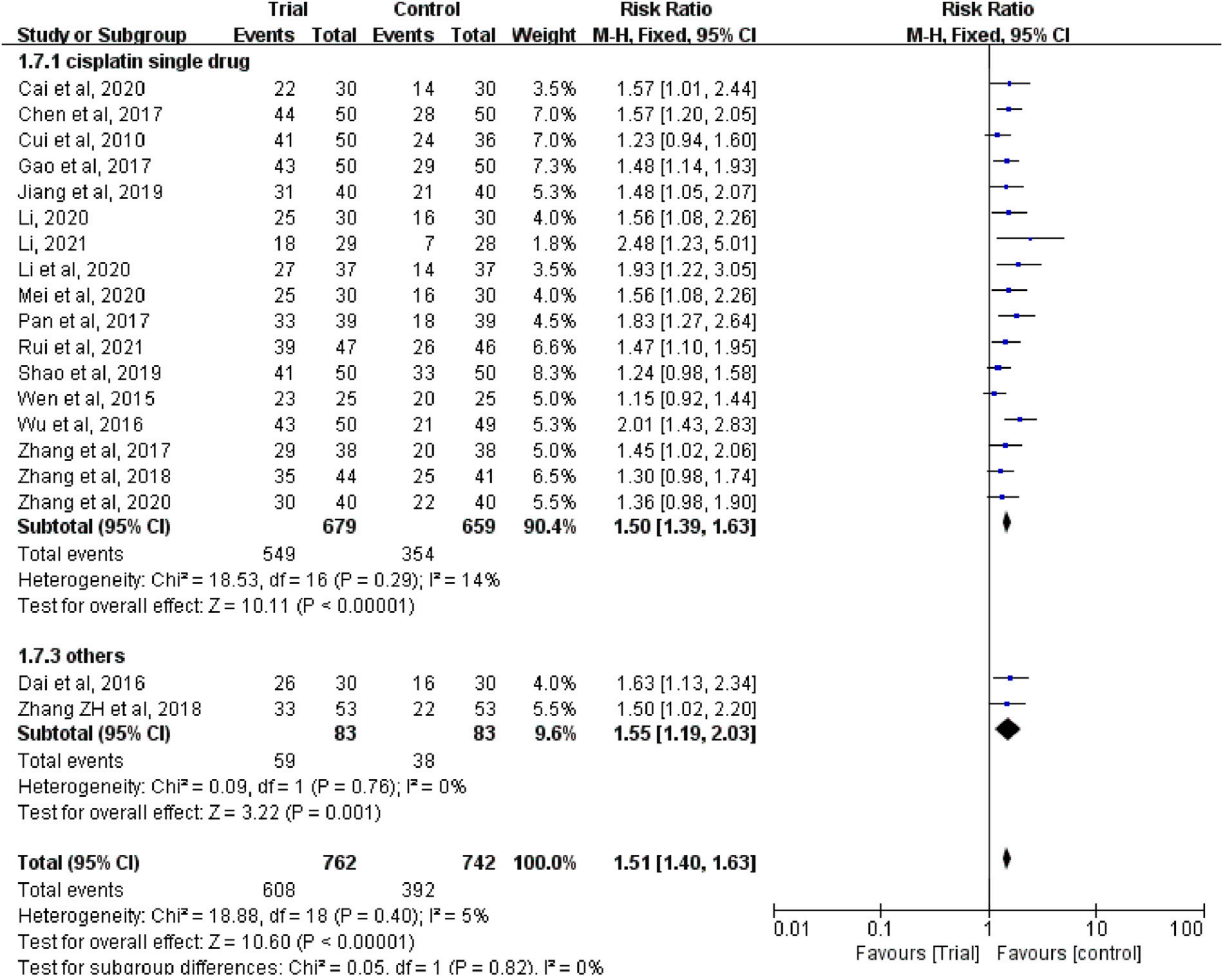
Subgroup analysis of clinical efficacy according to different drugs for hyperthermic intraperitoneal chemotherapy.

**FIGURE 6:**
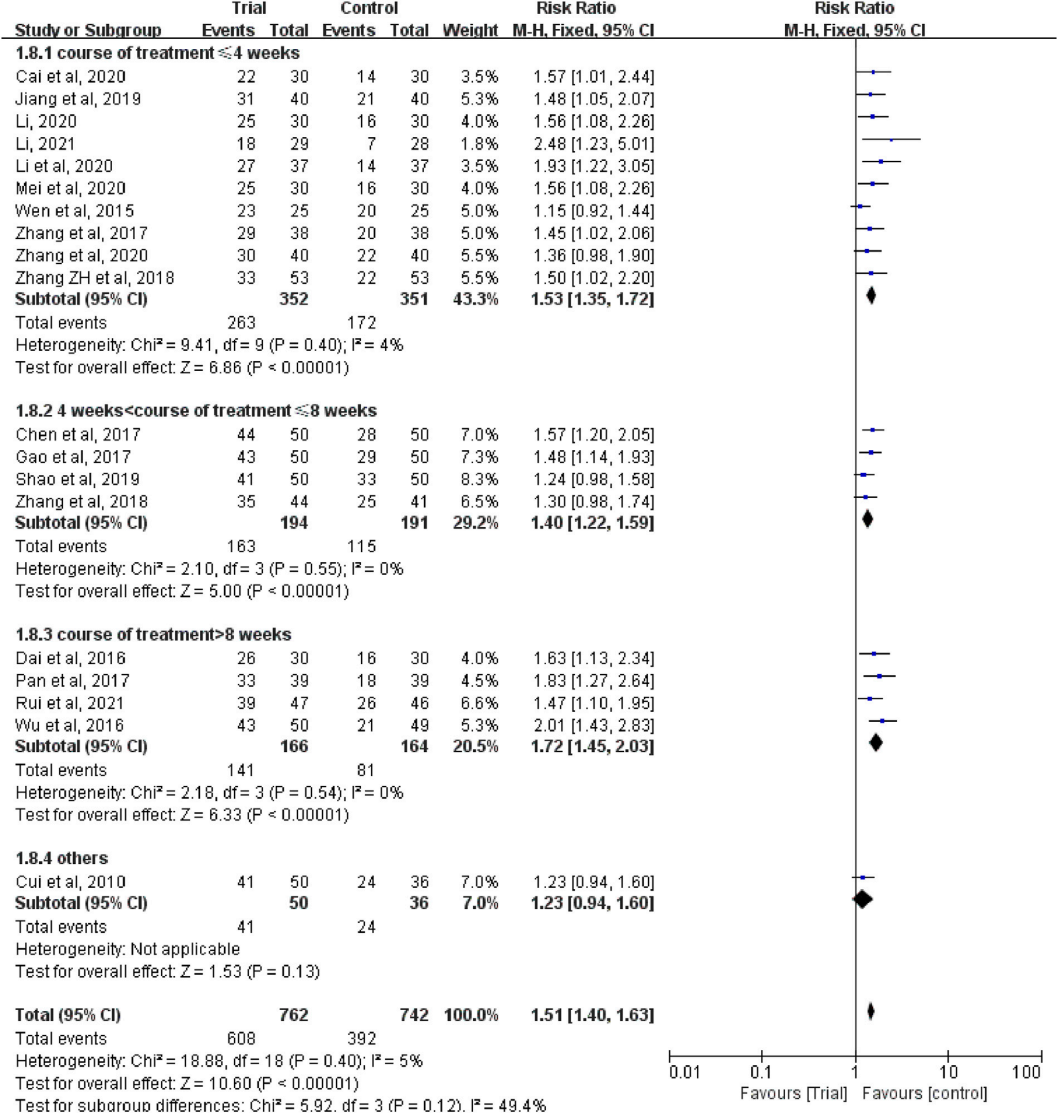
Subgroup analysis of clinical efficacy according to different course of treatment.

In these studies, a subgroup analysis of clinical efficacy was performed according to whether or not it was combined with intravenous chemotherapy (Figure 3). The results showed that the heterogeneity was increased in the subgroup with intravenous chemotherapy (I^2^ = 46%) and the heterogeneity was decreased in the subgroup without intravenous chemotherapy (I^2^ = 0%), but with or without intravenous chemotherapy, the clinical efficacy of the trial group was better than that of the control group (RR = 1.52, 95% CI [1.33, 1.73], *p* < 0.00001; RR = 1.5, 95% CI [1.37, 1.65], *p* < 0.00001). A subgroup analysis of clinical efficacy was performed according to different ways of using TCM (oral or external) (Figure 4). The results showed that the heterogeneity was decreased (I^2^ = 0%) in the subgroup of oral administration of TCM (I^2^ = 0%) and heterogeneity was increased (I^2^ = 43%) in the subgroup of external application of TCM, but regardless of whether the TCM was used orally or externally, the clinical efficacy of the trial group was better than that of the control group (RR = 1.53, 95% CI [1.41, 1.67], *p* < 0.00001; RR = 1.41, 95% CI [1.20, 1.67], *p* < 0.0001). We also performed a subgroup analysis of clinical efficacy according to different drugs for HIPEC (cisplatin single drug or others) (Figure 5). The results showed that heterogeneity was increased in the subgroup of receiving cisplatin single drug for HIPEC (I^2^ = 14%) and decreased in the subgroup of receiving HIPEC without cisplatin single drug (I^2^ = 0%), but the clinical efficacy of the trial group was better than that of the control group no matter what kind of chemotherapy drugs were used for HIPEC (RR = 1.5, 95% CI [1.39, 1.63], *p* < 0.00001; RR = 1.55, 95% CI [1.19, 2.03], *p* = 0.001). In addition, we also performed subgroup analysis of clinical efficacy according to the different course of treatment (course of treatment ≤ 4 weeks or 4 weeks < course of treatment ≤ 8 weeks or course of treatment > 8 weeks) (Figure 6). The results showed that the heterogeneity was basically unchanged in the subgroup with a course of treatment ≤4 weeks (I^2^ = 4%). The heterogeneity was decreased in the subgroups with 4 weeks < course of treatment ≤8 weeks and course of treatment >8 weeks (I^2^ = 0%). When we analyzed the clinical efficacy of these three subgroups, we found that the clinical efficacy of the trial group was better than that of the control group (RR = 1.53, 95% CI [1.35, 1.72], *p* < 0.00001; RR = 1.4, 95% CI [1.22, 1.59], *p* = 0.00001; RR = 1.72, 95% CI [1.45, 2.03], *p* < 0.00001).

### Ascites volume

**FIGURE 7:**
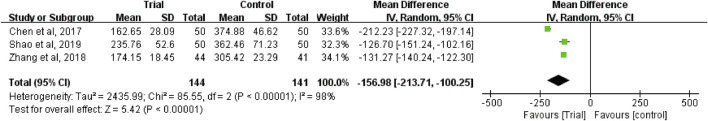
Forest plots of ascites volume.

Three studies (Chen and Hua, 2017; Zhang et al., 2018; Shao et al., 2019) reported the ascites volume with a total of 285 patients, including 144 patients in the trial group and 141 patients in the control group. Owing to heterogeneity between studies (I^2^ = 98%, *p* < 0.00001), a random-effect model was used. The results showed that TCM combined with HIPEC could effectively reduce the ascites volume (MD = −156.98, 95% CI [−213.71, −100.25], *p* < 0.00001, Figure 7).

### Abdominal circumference

**FIGURE 8:**
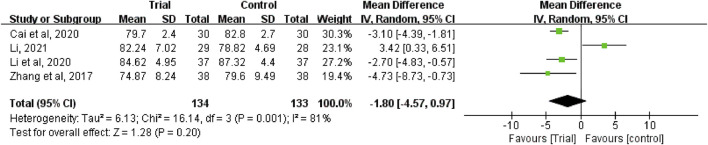
Forest plots of abdominal circumference.

Abdominal circumference data can be clearly extracted from four studies (Zhang et al., 2017; Cai and Zhu, 2020; Li et al., 2020; Li, 2021), with a total of 267 patients, including 134 patients in the trial group and 133 patients in the control group. Due to heterogeneity between studies (I^2^ = 81%, *p* = 0.001), a random-effect model was used. The results showed that the result was not statistically significant (MD = −1.8, 95% CI [−4.57, −0.97], *p* = 0.2, Figure 8).

### Karnofsky performance status

**FIGURE 9:**
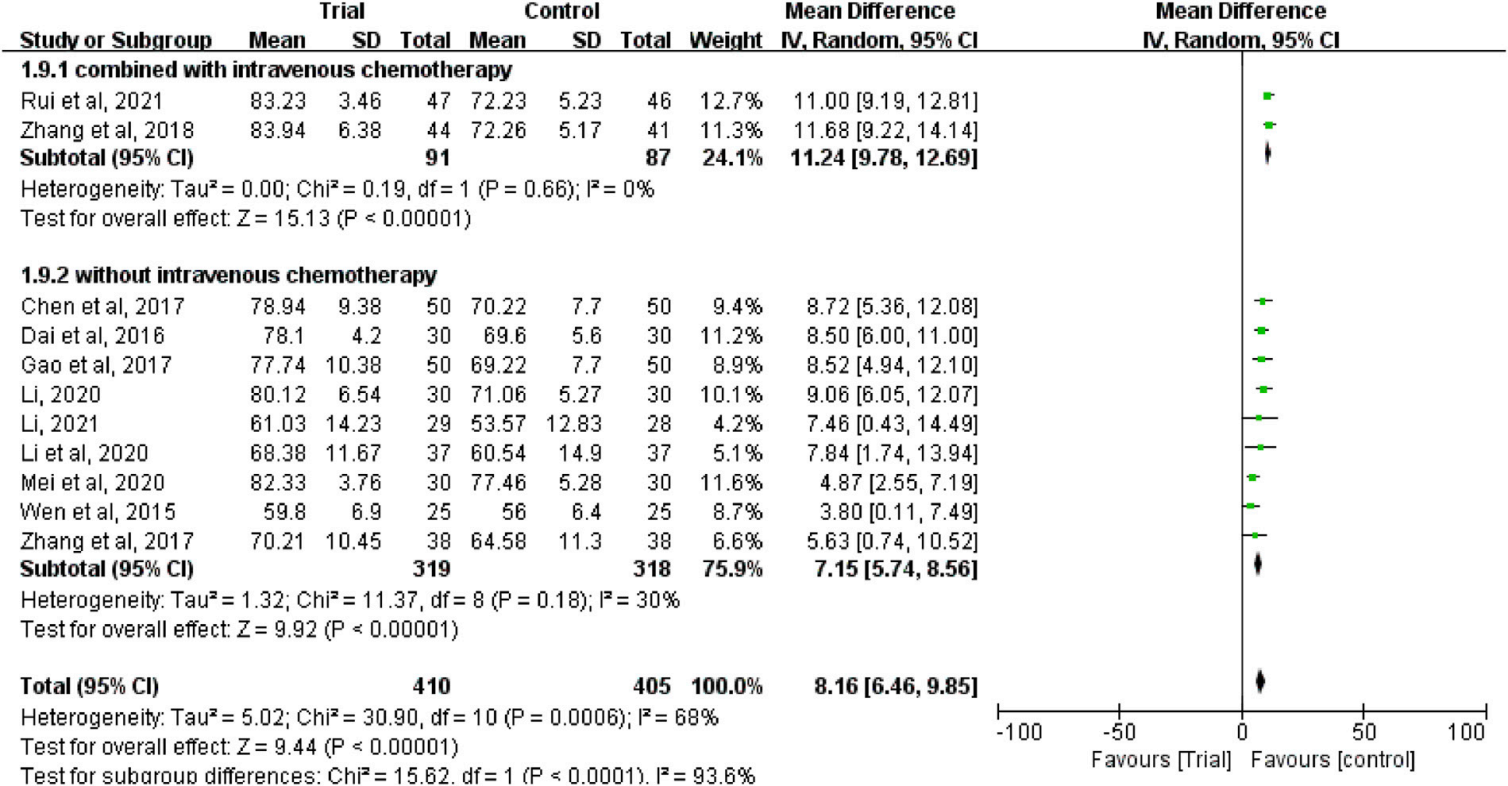
Subgroup analysis of the KPS according to whether combined with intravenous chemotherapy.

Eleven studies (Wen et al., 2015; Dai et al., 2016; Chen and Hua, 2017; Gao and Zhang, 2017; Zhang et al., 2017; Zhang et al., 2018; Li, 2020; Li et al., 2020; Mei et al., 2020; Li, 2021; Rui and Peng, 2021) reported the KPS score with a total of 815 patients, including 410 patients in the trial group and 405 patients in the control group. Due to heterogeneity between studies (I^2^ = 68%, *p* = 0.0006), a random-effect model was used. The results showed that the KPS score of TCM combined with HIPEC was significantly higher than that of single HIPEC (MD = 8.16, 95% CI [6.46, 9.85], *p* < 0.00001, Figure 9).

**FIGURE 10:**
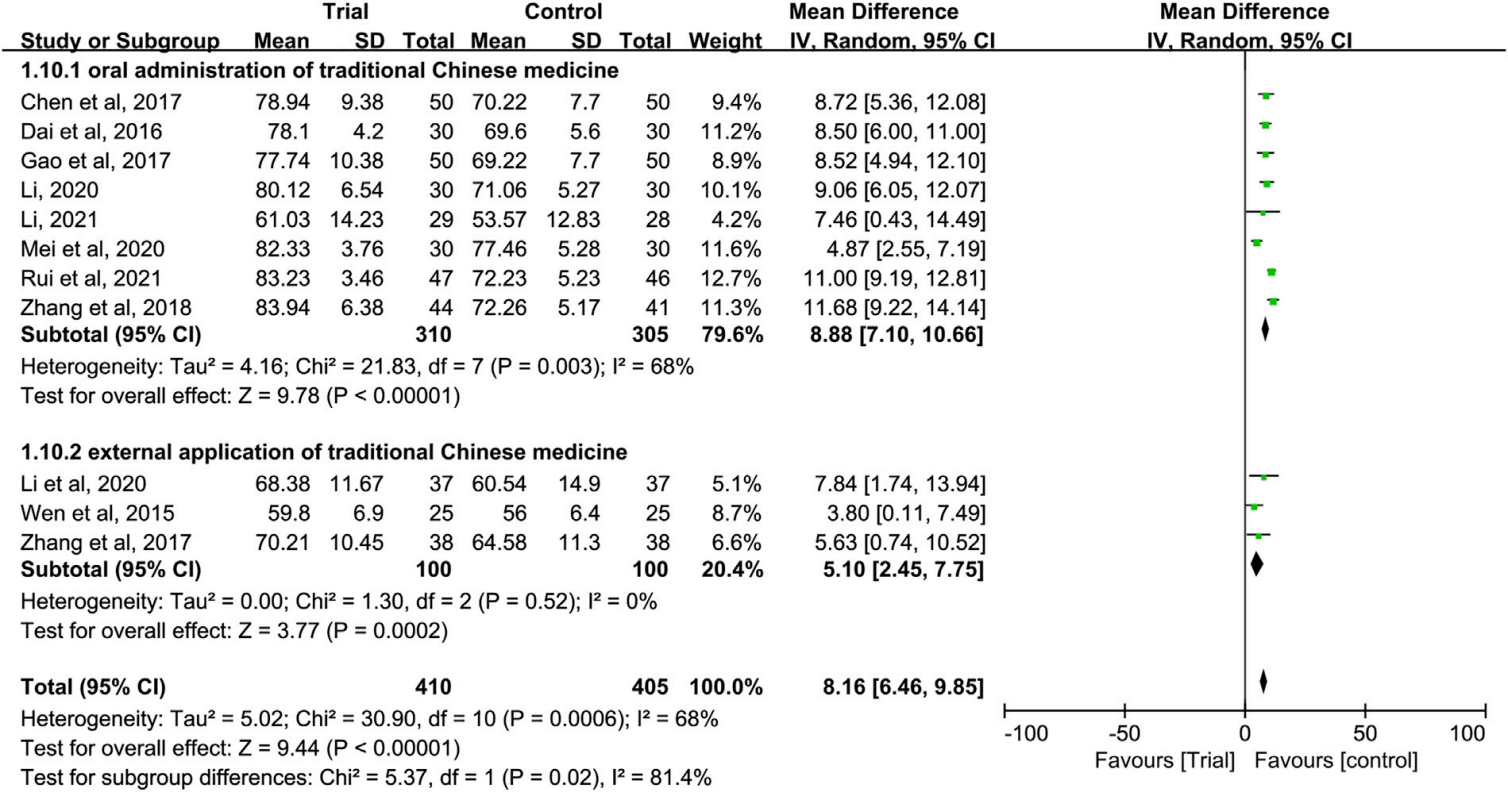
Subgroup analysis of the KPS according to different ways of using traditional Chinese medicine.

**FIGURE 11:**
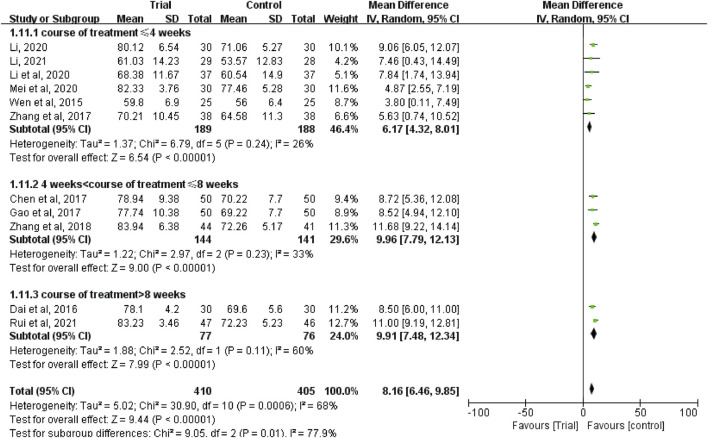
Subgroup analysis of the KPS according to different course of treatment.

In these studies, subgroup analysis of KPS score was performed according to whether or not intravenous chemotherapy was combined (Figure 9). The results showed that the heterogeneity of the two groups was decreased (I^2^ = 0%; I^2^ = 30%), and KPS score of the trial group was higher than that of the control group (MD = 11.24, 95% CI [9.78, 12.69], *p* < 0.00001; MD = 7.15, 95% CI (5.74 to 8.56], *p* < 0.00001). A subgroup analysis of KPS score was performed according to different ways of using TCM (oral or external) (Figure 10). The results showed that the heterogeneity remained unaffected (I^2^ = 68%) in the subgroup of oral administration of TCM (I^2^ = 68%). Heterogeneity was decreased in the subgroup of external application of TCM (I^2^ = 0%), but no matter whether the TCM was used orally or externally, the KPS score of the trial group were higher than that of the control group (MD = 8.88, 95% CI [7.10, 10.66], *p* < 0.00001; MD = 5.10, 95% CI [2, 45, 7.75], *p* = 0.0002). In addition, we also performed subgroup analysis of KPS score according to the different course of treatment (course of treatment ≤4 weeks or 4 weeks < course of treatment ≤8 weeks or course of treatment >8 weeks) (Figure 11). The results showed that the heterogeneity was decreased in the subgroups with the course of treatment ≤ 4 weeks and 4 weeks < course of treatment ≤8 weeks (I^2^ = 26%; I^2^ = 33%). Heterogeneity was basically unchanged in the subgroup with a course of treatment >8 weeks (I^2^ = 60%). When we analyzed the clinical efficacy of these three subgroups, we found that the KPS score of the trial group was higher than that of the control group (MD = 6.17, 95% CI [4.32, 8.01], *p* < 0.00001; MD = 9.96, 95% CI [7.79, 12.13], *p* < 0.00001; MD = 9.91, 95% CI [7.48, 12.34], *p* < 0.00001).

### Adverse events

Of the 19 included studies, 16 studies (Cui and Wu, 2010; Wen et al., 2015; Dai et al., 2016; Wu et al., 2016; Chen and Hua, 2017; Gao and Zhang, 2017; Pan et al., 2017; Zhang et al., 2017; Zhang et al., 2018; Jiang and Hu, 2019; Shao et al., 2019; Li, 2020; Li et al., 2020; Mei et al., 2020; Zhang et al., 2020; Rui and Peng, 2021) reported adverse events. Among them, patients of 14 studies (Cui and Wu, 2010; Dai et al., 2016; Wu et al., 2016; Chen and Hua, 2017; Gao and Zhang, 2017; Pan et al., 2017; Zhang et al., 2017; Zhang et al., 2018; Jiang and Hu, 2019; Shao et al., 2019; Li et al., 2020; Mei et al., 2020; Zhang et al., 2020; Rui and Peng, 2021) in both trial group and the control group developed myelosuppression and gastrointestinal reaction. Patients of six studies (Cui and Wu, 2010; Chen and Hua, 2017; Gao and Zhang, 2017; Jiang and Hu, 2019; Shao et al., 2019; Rui and Peng, 2021) in both trial group and control group developed hepatic dysfunction, and some patients in both trial group and control group experienced adverse reactions such as renal dysfunction or alopecia, but further research found that the probability of adverse events in the trial group was generally less than that in the control group. In three studies (Zhang et al., 2018; Li, 2020; Mei et al., 2020), the patients in the control group had more adverse reactions such as hepatic and renal dysfunction, neurotoxicity, cardiotoxicity and fever than those in the trial group. In addition, only one study (Zhang et al., 2017) showed rash symptoms in the trial group. One study (Wen et al., 2015) did not mention specific adverse events. Three studies (Zhang and Li, 2018; Cai and Zhu, 2020; Li, 2021) did not mention adverse events.

### Publication bias and sensitivity analysis

**FIGURE 12:**
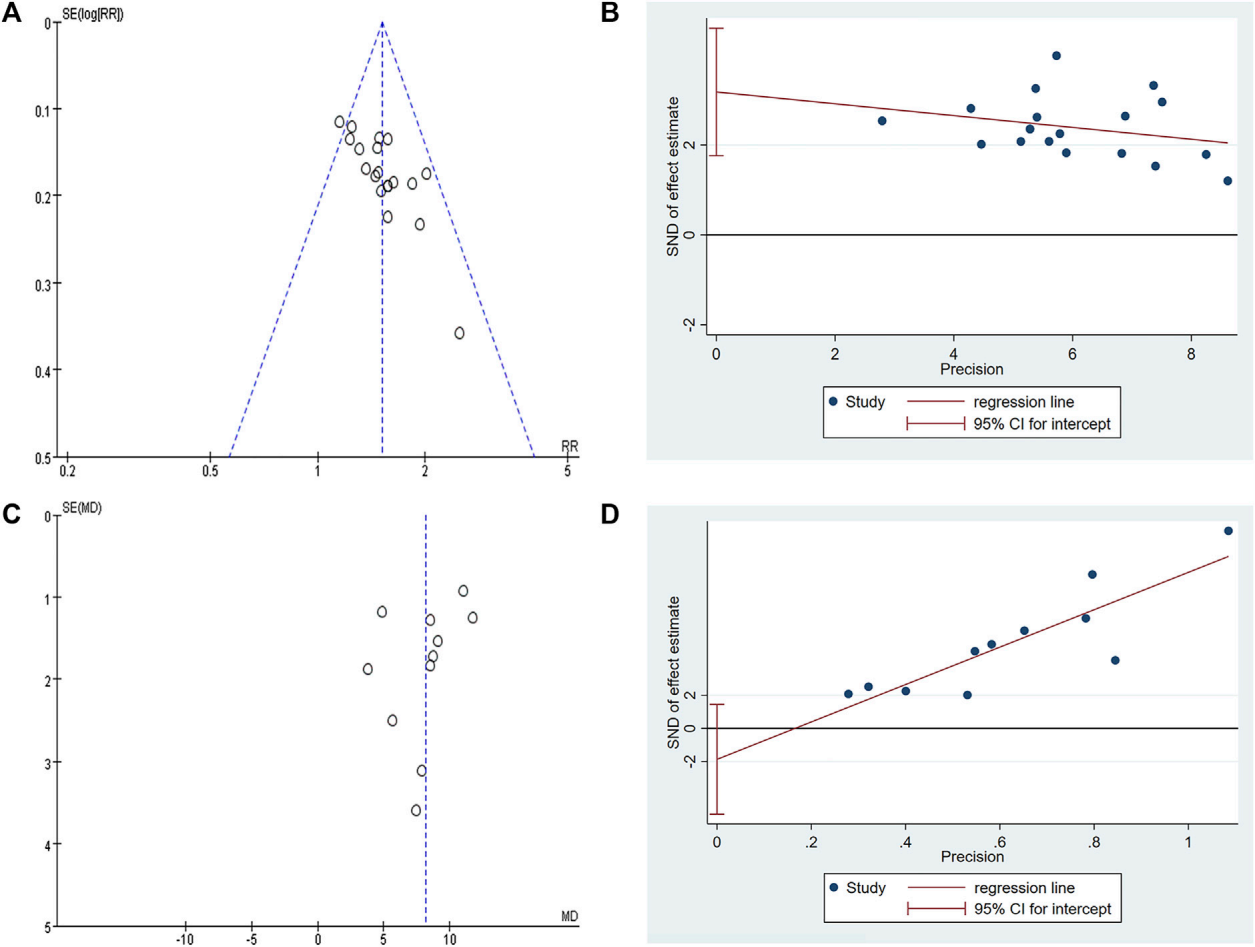
Publication bias plots. **(A)** Funnel plot of clinical efficacy; **(B)** Egger’s plot of clinical efficacy; **(C)** Funnel plot of KPS; **(D)** Egger’s plot of KPS.

The funnel plot of clinical efficacy (Figure 12A) was visually asymmetrically distributed, which might suggests publication bias. Egger’s test results (Figure 12B) were consistent with the funnel plot results (t = 4.73, *p* < 0.001), further proving the existence of publication bias in clinical efficacy. The KPS funnel plot (Figure 12C) was visually asymmetrically distributed, but the Egger’s test result (Figure 12D) was contrary (t = −1.27, *p* = 0.234), indicating that there was no obvious publication bias in the KPS studies. Since the number of RCTs included in the two studies of abdominal circumference and abdominal volume was both less than 10, the funnel plot-based publication bias test and Egger’s test were not performed. Sensitivity analyses of clinical efficacy, ascites volume and KPS were performed. After we excluded each study one by one, the results did not change significantly, indicating good stability of clinical efficacy, ascites volume, and KPS outcome data. However, when we performed a sensitivity analysis on abdominal circumference, we found that after excluding one study (Li, 2021), the results changed significantly, with P from 0.2 to <0.00001 and I^2^ from 81% to 0%.

## Discussion

This study systematically analyzed the clinical evidence of TCM combined with HIPEC in the treatment of MA, so as to better guide clinical practice. Analysis of 19 RCTs showed that TCM combined with HIPEC had a significant effect on the treatment of MA, which could reduce ascites volume and improve KPS score. The results of the combined analysis of ascites volume showed that there was heterogeneity among different studies, but after excluding the study of Chen and Hua, 2017, we found that the heterogeneity became 0%, and the trial group could still effectively reduce ascites volume compared with the control group. This may be related to the fact that study of Chen and Hua, 2017 used cisplatin single agent for HIPEC, while the other two studies used paclitaxel intravenous chemotherapy combined with cisplatin HIPEC. In addition, the results of the combined analysis of abdominal circumference showed that there was heterogeneity among studies, but after excluding the study of Li, 2021, we found that the heterogeneity was decreased to 0%, and the trial group could effectively reduce the abdominal circumference compared with the control group. We analyzed the possible reasons as follows: 1) Some tumor patients might have a huge abdominal mass, which might cause the measurement of the abdominal circumference, thus affecting the trial data; 2) The study of Li, 2021 included patients diagnosed as spleen-kidney yang deficiency in TCM syndrome, which was different from the inclusion criteria of other studies. What’s more, we also found that there was heterogeneity in the analysis results of KPS score. Therefore, we conducted a subgroup analysis based on whether combined with intravenous chemotherapy or not, and the results showed that subgroup heterogeneity was significantly reduced, indicating that whether or not it was combined with intravenous chemotherapy is one of the important factors affecting heterogeneity. According to the subgroup analysis of different ways of using TCM, the results showed that the subgroup heterogeneity of oral administration of TCM did not change, while the subgroup heterogeneity of external application of TCM decreased dramatically. We speculated that it might be because the drugs for HIPEC in the subgroup of external application of TCM were cisplatin single drug, while the subgroup of oral administration of TCM used different drugs for HIPEC or combined with intravenous chemotherapy, resulting in the heterogeneity of this subgroup. According to the course of treatment, subgroup analysis showed that the heterogeneity of the treatment course ≤4 weeks and 4 weeks < treatment course ≤8 weeks decreased significantly, while the heterogeneity of the treatment course >8 weeks remained basically unchanged, which might be related to the small number of studies included in the subgroup >8 weeks. Combining the three subgroups analyses of the KPS score, the results showed that whether combined with intravenous chemotherapy, different ways of using TCM (oral or external) and different course of treatment were important factors influencing the heterogeneity of the results. In addition, our analysis found that the heterogeneity of clinical efficacy in each subgroup was less than 50%, and the trial group could significantly improve the clinical efficacy compared with the control group. The consistency of these results enhanced the reliability of the conclusion that TCM combined with HIPEC can effectively treat MA. Among the 19 included studies, 16 studies reported adverse events. After our observation and analysis, we believed that these adverse events might be caused by chemotherapy drugs, further analysis found that the probability of adverse events in the trial group was equal to or even lower than that in the control group, it could be considered that TCM combined with HIPEC might be safe. The results of visual evaluation of funnel plot and Egger’s test showed that there was publication bias in clinical efficacy. We believed that the possible reasons were as follows: 1) The sample size of the included RCTs was small; 2) Lack of negative results; 3) Except for six studies (Dai et al., 2016; Zhang et al., 2017; Jiang and Hu, 2019; Mei et al., 2020; Zhang et al., 2020; Rui and Peng, 2021) reported the sources of funding, other studies did not clearly report the sources of funding, and it was uncertain whether there was a conflict of interest. In the sensitivity analysis of abdominal circumference, we found that heterogeneity was decreased significantly after excluding a study of Li, 2021, and the results were statistically significant. The inconsistency of the results might be due to the inclusion of patients diagnosed as spleen-kidney yang deficiency in TCM syndrome in this study, which was different from other studies. It was also possible that the small sample size of included studies made the results unstable. In addition, the statistical analysis of the frequency and medicinal properties of herbs for the treatment of MA found that the frequency of qi-invigorating herbs was the highest, because MA is a symptom of deficiency in origin and excess in superficiality, and deficiency of qi leads to stagnation of qi, and qi deficiency is the root. The application of qi-invigorating herbs to promote qi can promote the operation of water and liquid, which is conducive to the elimination of MA. Herbs for inducing diuresis and excreting were second. In the treatment of MA, no matter whether it was deficient or excessive, no matter what type of syndrome it was, it was necessary to add herbs for inducing diuresis and excreting in the prescription to promote the discharge of water. From the analysis of the frequency of use of a single herb, the frequency of commonly used herbs was basically consistent with the properties of the herbs, suggesting the importance of qi-invigorating herbs in the treatment of MA, and most of the herbs that have both qi-invigorating and diuresis-promoting effects were used.

MA is mainly caused by intra-abdominal metastasis of advanced malignant tumors, and the disease is dangerous, with poor quality of life and short survival. Currently, there is not any standard treatment plan. Although the survival of patients with MA is limited, successful palliative treatment can greatly improve the prognosis of patients (Levine et al., 2007). Modern medical research believes that MA is the result of a combination of multiple factors, and the main mechanism can be summarized as the reduction of lymphatic reflux absorption and excessive fluid production (He et al., 2013). At present, WM treatment mainly starts from the treatment of primary disease and the treatment of MA, including restriction of water and sodium intake, use of diuretics, drainage of MA, and intravenous or intraperitoneal infusion of chemotherapy drugs (Cavazzoni et al., 2013). In recent years, anti-angiogenic drugs had been used in many studies to treat MA (Hsu et al., 2019), and Alfapump system had also been used for continuous low-flow ascites drainage through the bladder to reduce the need for repeated puncture (Fotopoulou et al., 2019). However, these treatments have limitations, such as high side effects of drugs, poor tolerance of treatment by patients, ow feasibility of promotion, and many factors can bring more difficulties. HIPEC is an emerging method for the treatment of MA in recent years, which can control the metastasis of cancer cells through the combination of intraperitoneal perfusion chemotherapy and hyperthermia (Turrini et al., 2012). Its principle is to perfuse the heated chemotherapy perfusate into the abdominal cavity, so that the intra-abdominal temperature rises to the effective treatment temperature and maintains it for a certain period of time. It promotes a large amount of heat absorption in the visceral peritoneum, parietal peritoneum and arterial beds of various organs in the abdominal cavity, using the difference in temperature tolerance between tumor cells and normal cells, it can cause the destruction of tumor blood vessels and block the blood supply of tumor cells, thereby inhibiting the proliferation of tumor cells and increasing the sensitivity of patients to chemotherapy drugs, so as to achieve the therapeutic purpose of inducing tumor cell apoptosis and effectively killing tumor cells without damaging normal cells (Cui et al., 2012; Glehen et al., 2014). In addition, studies have confirmed that HIPEC also has a significant effect on inhibiting the expression level of drug-resistant genes in cancer cells (Zhang et al., 2014). However, drugs for HIPEC have poor penetration, which can only act 2 mm below the tumor surface, and the adverse reactions such as gastrointestinal reaction, leukopenia and water and electrolyte disorders are inevitable during the treatment process (Li et al., 2012). TCM classifies MA in the category of tympanites, which belongs to dropsy in tympanites. Its pathogenesis is dysfunction of the liver, spleen and kidney, and stagnantion of pneuma, blood stasis and water retention are interlinked in the abdomen. The pathological characteristics are deficiency in origin and excess in superficiality (Wu et al., 2015). The initial stage is liver stagnation and spleen deficiency, dysfunction of the spleen in transport, deficiency of the kidney essence, further development of the disease leads to the kidney yang deficiency, resulting in the spleen-kidney deficiency, water and moisture retention are even worse. The liver storing blood, kidney storing essence, homogeny of liver and kidney, stagnation of liver qi causing heat-transmission and consumption of yin. Liver yin deficiency for a long time will extend kidney, so that the yin deficiency of liver and kidney aggravates the distention (Zhang et al., 2019). This disease is locally excess, and the whole body is deficient. This primary deficiency is a deficiency of spleen-yang and kidney-yang, deficiency of liver-yin and kidney-yin, and the treatment should be based on the principles of reinforcement and elimination in combination as well as strengthening the body resistance (Wu et al., 2015). HIPEC is equivalent to TCM therapy for eliminating the pathogen. In order to prevent and cure the injury of human body’s vital qi caused by HIPEC and better achieve the purpose of strengthening the body resistance to eliminate pathogenic factors, it is necessary to take it orally or externally with TCM for syndrome differentiation and treatment. What’s more, studies have pointed out that Chinese herbs for strengthening the body’s resistance can enhance the activity of natural killer cells *in vivo*, thus enhancing cellular immunity in the body, and can eliminate the release of cytotoxins by cancer cells, thereby improving the life quality of patients (Peng and Su, 2017).

This study has certain limitations. First, the methodological quality of the included studies was not high, and they reported inadequate information on allocation sequence generation, allocation concealment, and blinding. Second, all the included studies were conducted in China, and no foreign countries were involved. Therefore, it is difficult to verify whether the efficacy of TCM combined with HIPEC in the treatment of MA is applicable to populations in other regions. Third, there might be heterogeneity among most of the included studies: 1) different characteristics of participants, such as age, gender, tumor type, etc.; 2) different temperature, course, drugs and dose of HIPEC; 3) the prescription composition and course of treatment of TCM were different. Fourth, there was a potential publication bias in the clinical efficacy of this study, which might affect the reliability of the study results. Fifth, although most studies reported adverse events, it was not clear whether the adverse events were caused by HIPEC or TCM. In addition, some studies did not report data on adverse events in the trial and control groups, and some studies lacked data that could be analyzed. Despite these limitations, this study is the first to systematically evaluate the efficacy of TCM combined with HIPEC in MA, which may be helpful to clinicians.

## Conclusion

The results of this study indicated that TCM combined with HIPEC can not only improve the efficacy of MA, but also improve the quality of life of patients. However, due to the small sample size and the risk of bias of included studies, further standard, double-blind, multicenter RCTs are needed to confirm the clinical value of TCM combined with HIPEC in the treatment of MA. In addition, whether the ways of using TCM will affect the clinical efficacy of MA is the focus of future research.

## Data Availability

The original contributions presented in the study are included in the article/Supplementary Material, further inquiries can be directed to the corresponding author.

## Abbreviations

CNKI, China national knowledge infrastructure; CI, confidence interval; CR, complete remission; HIPEC, hyperthermic intraperitoneal chemotherapy; I^2^, I-square; KPS, karnofsky performance status; MA, malignant ascites; MD, mean difference; PD, progression disease; PR, partial remission; RCTs, randomized controlled trials; RR, risk ratio; SD, stable disease; TCM, traditional Chinese medicine; VIP, Chinese scientific journal database; WM, western medicine.
