# Supplementary material for: The efficacy of traditional chinese medicine combined with hyperthermic intraperitoneal chemotherapy for malignant ascites: A systematic review and meta-analysis
Source: Front Pharmacol. 2022 Aug 29;13:938472. doi: 10.3389/fphar.2022.938472 (PMC9467275; doi:10.3389/fphar.2022.938472)
Supplement: Supplementary file 1 [file Table1.docx]

**Supplement 1 shows the search strategy used for the systematic review**

Search strategy for PubMed

((traditional chinese medicine) OR (herbs)) AND ((hyperthermic intraperitoneal chemotherapy[Title/Abstract]) OR (peritoneal hyperthermic perfusion chemotherapy[Title/Abstract])) AND ((malignant ascites[Title/Abstract]) OR (malignant peritoneal effusion[Title/Abstract])). 0 literature was searched.

Search strategy for Cochrane Library

Date Run: 20/03/2022 19:56:37

Comment:

ID Search Hits

#1 (hyperthermic intraperitoneal chemotherapy) OR (peritoneal hyperthermic perfusion chemotherapy) 489

#2 (malignant ascites) OR (malignant peritoneal effusion) 348

#3 (traditional chinese medicine) OR (herbs) 22202

#4 #1 AND #2 AND #3 2

Search strategy for Embase

# ▲ 检索内容

1 (traditional chinese medicine or herbs).af. 132230

2 (hyperthermic intraperitoneal chemotherapy or peritoneal hyperthermic perfusion chemotherapy).ti. 2203

3 (malignant ascites or malignant peritoneal effusion).ab. 2115

4 1 and 2 and 3 0

Search strategy for China National Knowledge Infrastructure (CNKI)

TI='热灌注' AND AB=('腹水'+'腹腔积液') AND FT=('中医'+'中药'+'草药'). 108 literatures were searched.

Search strategy for Wanfang Database

题名:(热灌注) and 摘要:("腹水" or "腹腔积液") and 全部:("中医" or "中药" or "草药"). 86 literatures were searched.

Search strategy for Chinese Scientific Journal Database (VIP)

T=热灌注 * R=(腹水 + 腹腔积液) * U=(中医 + 中药 + 草药). 69 literatures were searched.

Search strategy for Sinomed

("腹水"[摘要:智能] OR "腹腔积液"[摘要:智能]) AND "热灌注"[标题:智能] AND ("中药"[全部字段:智能] OR "中医"[全部字段:智能] OR "草药"[全部字段:智能]). 71 literatures were searched.
