# Supplementary material for: The efficacy of traditional chinese medicine combined with hyperthermic intraperitoneal chemotherapy for malignant ascites: A systematic review and meta-analysis
Source: Front Pharmacol. 2022 Aug 29;13:938472. doi: 10.3389/fphar.2022.938472 (PMC9467275; doi:10.3389/fphar.2022.938472)
Supplement: Supplementary file 2 [file Table2.docx]

| Study | Source | Species, concentration | Quality control reported? (Y/N) | Chemical analysis reported? (Y/N) |
| --- | --- | --- | --- | --- |
| Rui et al, 2021 | Hefei Cancer Hospital, Chinese Academy of Sciences | Codonopsis pilosula (Franch.) Nannf. [Campanulaceae], 20g, Astragalus membranaceus (Fisch.) Bunge. [Leguminosae], 20g, Atractylodes macrocephala Koidz. [Compositae], 15g, Ligusticum chuanxiong Hort. [umbelliferae], 15g，Agrimonia pilosa Ldb. [Rosaceae], 15g, Curcuma phaeocaulis Valeton [Zingiberaceae], 15g, Hedyotis diffusa Willd [rubiaceae], 15g, Solanum nigrum L. [Solanaceae], 15g, Poria cocos(Schw.)Wolf [polyporaceae], 15g, Glehnia littoralis Fr. Schmidt ex Miq. [umbelliferae], 15g, Fructus Ligustri Lucidi [Oleaceae], 15g, Ophiopogon japonicus(Linn. f.) Ker-Gawl. [liliaceae], 15g, Polygonatum sibiricum Delar. ex Redoute [liliaceae], 15g, Glycyrrhiza uralensis Fisch. [Leguminosae], 10g | Y - Hospital preparations | N |
| Pan et al, 2017 | Xinhui District Hospital of Traditional Chinese Medicine, Jiangmen city, Guangdong Province | Panax quiquefolium L. [Araliaceae], 10g, Angelica sinensis (Oliv.) Diels [umbelliferae], 10g, Curcuma phaeocaulis Valeton [Zingiberaceae], 10g, Amomum villosum Lour. [Zingiberaceae], 10g, Astragalus membranaceus (Fisch.) Bunge. [Leguminosae], 15g, Cremastra appendiculata (D. Don) Makino [Orchidaceae], 15g, Atractylodes macrocephala Koidz. [Compositae], 15g, Glabrous Greenbrier Rhizome [liliaceae], 15g, Hedyotis diffusa Willd [rubiaceae], 8g, Scutellaria barbataD.Don [Lamiaceae], 8g | Y - Hospital preparations | N |
| Wu et al, 2016 | Hebei Chengde County Hospital | Scutellaria barbataD.Don [Lamiaceae], 15g, Cremastra appendiculata (D. Don) Makino [Orchidaceae], 15g, Glabrous Greenbrier Rhizome [liliaceae], 15g, Curcuma phaeocaulis Valeton [Zingiberaceae], 5g, Hedyotis diffusa Willd [rubiaceae], 15g, Whitmania pigra Whitman [Hirudinidae], 2g, Atractylodes macrocephala Koidz. [Compositae], 15g, Angelica sinensis (Oliv.) Diels [umbelliferae], 7g, Amomum villosum Lour. [Zingiberaceae], 7g, Astragalus membranaceus (Fisch.) Bunge. [Leguminosae], 15g, Panax quiquefolium L. [Araliaceae], 7g | Y - Hospital preparations | N |
| Zhang et al, 2018 | Qinhuangdao Traditional Chinese Medicine Hospital | Aconitum carmichaelii Debx [Ranunculaceae], 20g, Poria cocos(Schw.)Wolf [polyporaceae], 15g, Cinnamomum cassia Presl. [Lauraceae], 20g, Pseudostellaria radix [caryophyllaceae], 25g, Atractylodes macrocephala Koidz. [Compositae], 15g, Zingiber officinale Roscoe [Zingiberaceae], 15g, Coix lacryma-jobi L.var.mayuen(Roman.)Stapf [Gramineae], 20g, polyporus [Polyporaceae], 15g, Alisma orientalis(Sam.)Juzep. [Alismaceae], 15g, E.brevicornum Maxim. [Berberidaceae], 15g, Cornus officinalis Sieb. et Zucc. [Cornaceae], 20g, Dioscorea polystachya Turczaninow [Dioscoreaceae], 15g, Cynanchum otophyllum Schneid. [Asclepiadaceae], 15g, Citrus reticulata Blanco [Rutaceae], 10g, Ziziphus jujuba Mill. [Rhamnaceae], 10g, Glycyrrhiza uralensis Fisch. [Leguminosae], 5g | Y - Hospital preparations | N |
| Shao et al, 2019 | Zhoushan Hospital of Traditional Chinese Medicine, Zhejiang Province | Astragalus membranaceus (Fisch.) Bunge. [Leguminosae], 25g, Atractylodes macrocephala Koidz. [Compositae], 15g, Panax ginseng C. A. Meyer [Araliaceae], 15g, Ligusticum chuanxiong Hort. [Umbelliferae], 15g, Piper nigrum L. [Piperaceae], 12g, AgrimoniapilosaLedeb. [Rosaceae], 12g, Curcuma phaeocaulis Valeton [Zingiberaceae], 12g, Hedyotis diffusa Willd [rubiaceae], 12g, Glycyrrhiza uralensis Fisch. [Leguminosae], 8g | Y - Hospital preparations | N |
| Li, 2020 | Hebei Chengde County Hospital | Astragalus membranaceus (Fisch.) Bunge. [Leguminosae], 30g, Atractylodes macrocephala Koidz. [Compositae], 30g, Gynostemma pentaphyllum (Thunb.) Makino [Cucurbitaceae], 30g, Dendrobium nobile Lindl. [Orchidaceae], 15g, Coix lacryma-jobi L.var.mayuen(Roman.)Stapf [Gramineae], 30g, Polyporus [Polyporaceae], 15g, Hedyotis diffusa Willd [rubiaceae], 30g, Actinidia arguta (Sieb. et Zucc) Planch. ex Miq. [Actinidiaceae], 30g, VitiswilsonaeVeitch [Vitaceae], 30g, Foreknowledge [Lardizabalaceae], 15g, Panax notoginseng (Burkill) F. H. Chen ex C. H. [Araliaceae], 15g, Gallusgallusdomesticus Brisson [Phasianidae], 10g, Glycyrrhiza uralensis Fisch. [Leguminosae], 10g | Y - Hospital preparations | N |
| Cui et al, 2010 | Xuchang Traditional Chinese Medicine Hospital | Poria cocos(Schw.)Wolf [polyporaceae], 12g, Cynanchum otophyllum Schneid. [Asclepiadaceae], 15g, Atractylodes macrocephala Koidz. [Compositae], 12g, Zingiber oj-jicinale Rosc. [Zingiberaceae], 12g, Aconitum carmichaelii Debx [Ranunculaceae], 10g, Astragalus membranaceus (Fisch.) Bunge. [Leguminosae], 30g, Areca Peel [Palmaceae], 15g, Alisma orientalis(Sam.)Juzep. [Alismaceae], 20g, Cuscuta chinensis Lam. [Convolvulaceae], 20g, Plantago asiatica L. [Plantaginaceae], 30 g, Rice sprout [Gramineae], 15g, Hordeurn vulgare L. [Gramineae], 15 g, Glycyrrhiza uralensis Fisch. [Leguminosae], 6 g | Y - Hospital preparations | N |
| Cai et al, 2020 | Yueyang Integrated Traditional Chinese and Western Medicine Hospital | Panax ginseng C. A. Meyer [Araliaceae], 9g, Atractylodes macrocephala Koidz. [Compositae], 9g, Coix lacryma-jobi L.var.mayuen(Roman.)Stapf [Gramineae], 15g, Poria cocos(Schw.)Wolf [polyporaceae], 15g, Platycodon grandiflorus(Jacq.)A.Dc. [Campanulaceae], 6g, Cuscuta chinensis Lam. [Convolvulaceae], 15g, Morinda ojj:icinalis How [Rubiaceae], 15g, E.brevicornum Maxim. [Berberidaceae], 15g, Ziziphus jujuba Mill. [Rhamnaceae], 10g, Euphorbia kansui T. N. Liou ex S. B. Ho [Euphorbiaceae], l5g, Daphne genkwa Sieb. et Zucc. [Thymelaeaceae], 15g, Hiraute Shiny Bugleweed Herb [Lamiaceae], 15g | Y - Hospital preparations | N |
| Li, 2021 | Anhui University of Traditional Chinese Medicine | Acanthopanar gracilistμlusW.W.Smith [Araliaceae], 10g, Atractylodes macrocephala Koidz. [Compositae], 15g, Areca Peel [Palmaceae], 15g, Citrus reticulata Blanco [Rutaceae], 30g, Poria cocos(Schw.)Wolf [polyporaceae], 30g, Aconitum carmichaelii Debx [Ranunculaceae], 6g, Astragalus membranaceus (Fisch.) Bunge. [Leguminosae], 30g, Alisma orientalis(Sam.)Juzep. [Alismaceae], 10g, Cinnamomum cassia Presl. [Lauraceae], 9g, Codonopsis pilosula (Franch.) Nannf. [Campanulaceae], 15g | Y - Hospital preparations | N |
| Jiang et al, 2019 | Yongkang Traditional Chinese Medicine Hospital of Zhejiang Province | Aconitum carmichaelii Debx [Ranunculaceae], 30g, Cinnamomum cassia Presl. [Lauraceae], 15g, Zingiber oj-jicinale Rosc. [Zingiberaceae], 15g, Morinda ojj:icinalis How [Rubiaceae], 15g, E.brevicornum Maxim. [Berberidaceae], 15g, Poria cocos(Schw.)Wolf [polyporaceae], 15g, Atractylodes macrocephala Koidz. [Compositae], 15g, Cynanchum otophyllum Schneid. [Asclepiadaceae], 15g, Panax ginseng C. A. Meyer [Araliaceae], 15g, Hedyotis diffusa Willd [rubiaceae], 15g, Curcuma phaeocaulis Valeton [Zingiberaceae], 10g, Sparganium stoloni erum,Buch. -Ham. [Sparganiaceae], 8g | Y - Hospital preparations | N |
| Chen et al, 2017 | Hangzhou Cancer Hospital | Poria cocos(Schw.)Wolf [polyporaceae], 20g, Zingiber officinale Roscoe [Zingiberaceae], 15g, Aconitum carmichaelii Debx [Ranunculaceae], 15g, Atractylodes macrocephala Koidz. [Compositae], 15g, Cynanchum otophyllum Schneid. [Asclepiadaceae], 15 g | Y - Hospital preparations | N |
| Mei et al, 2020 | Jiujiang Traditional Chinese Medicine Hospital | Rehmannia glutinosa (Gaert.) Libosch. ex Fisch. et Mey. [Scrophulariaceae], 30g, Anemarrhena asphodeloides Bunge [Liliaceae], 20g, Cornus officinalis Sieb. et Zucc. [Cornaceae], 30g, Dioscorea polystachya Turczaninow [Dioscoreaceae], 30g, Poria cocos(Schw.)Wolf [polyporaceae], 20g, E.brevicornum Maxim. [Berberidaceae], 20g, Bombyx mori Linnaeus [Bombycidae], 20g, Trionyx sinensis Wiegmann [Trionychidae], 20g, Manis [Manidae], 20g, Salvia miltiorrhiza Bge. [Lamiaceae], 20g, Lindera aggregata(Sims) Kosterm. [Lauraceae], 30g, Folium Hibisci Mutabilis [Malvaceae], 20g, Prunella vulgaris L [Lamiaceae], 30g, Phellodendron chinense Schneid. [Rutaceae], 20g | Y - Hospital preparations | N |
| Dai et al, 2016 | Hunan University of TCM | Aconitum carmichaelii Debx [Ranunculaceae], 9g, Poria cocos(Schw.)Wolf [polyporaceae], 20g, Zingiber officinale Roscoe [Zingiberaceae], 10g, Atractylodes macrocephala Koidz. [Compositae], 10g, Cynanchum otophyllum Schneid. [Asclepiadaceae], 10 g | Y - Hospital preparations | N |
| Zhang et al, 2020 | The Third Affiliated Hospital of Henan University of TCM | Glycyrrhiza uralensis Fisch. [Leguminosae], 8g, Artemisia capillaris Thunb. [Compositae], 12g, Taraxacum mongolicum Hand.-Mazz. [Compositae], 12g, Citrus aurantium L. [Rutaceae], 15g, Alisma orientalis(Sam.)Juzep. [Alismaceae], 15g, Polyporus [Polyporaceae], 18g, Areca Peel [Palmaceae], 18g, Salvia miltiorrhiza Bge. [Lamiaceae], 18g, Pseudostellaria radix [caryophyllaceae], 25g, Atractylodes macrocephala Koidz. [Compositae], 30g, Poria cocos(Schw.)Wolf [polyporaceae], 28g | Y - Hospital preparations | N |
| Gao et al, 2017 | Xi 'an Fifth Hospital, Shaanxi Province | Poria cocos(Schw.)Wolf [polyporaceae], 20g, Zingiber officinale Roscoe [Zingiberaceae], 10g, Atractylodes macrocephala Koidz. [Compositae], 10g, Cynanchum otophyllum Schneid. [Asclepiadaceae], 10g, Sophora flavescens Alt. [Leguminosae], 10g, Aconitum carmichaelii Debx [Ranunculaceae], 8g | Y - Hospital preparations | N |
| Zhang et al, 2017 | Anhui Provincial Hospital | Pheretima [Earthworm family], Buthus martensii Karsch [Buthidae], Strychni Semen [Loganiaceae], Toad [Bufonidae], Realgar, Sinapis alba L. [Cruciferae], Euphorbia kansui T. N. Liou ex S. B. Ho [Euphorbiaceae], Daphne genkwa Sieb. et Zucc. [Thymelaeaceae], Areca Peel [Palmaceae], Pinellia ternata( Thunb.) Breit. [Araceae], Rheum palmatum L. [Polygonaceae], Salvia miltiorrhiza Bge. [Lamiaceae]^a^ | Y - Hospital preparations | N |
| Zhang ZH et al, 2018 | Integrated Traditional Chinese and Western Medicine Hospital of Southern Medical University | Polyporus [Polyporaceae], Astragalus membranaceus (Fisch.) Bunge. [Leguminosae], Carthamus tinctorius L. [Compositae], Plantago asiatica L. [Plantaginaceae], Prunus persica(L.)Batsch [Rosaceae], Coix lacryma-jobi L.var.mayuen(Roman.)Stapf [Gramineae]^a^ | Y - Hospital preparations | N |
| Li et al, 2020 | Wuhu Hospital of Traditional Chinese Medicine | Aconitum carmichaelii [Ranunculaceae], 10g, Cinnamomum cassia Presl. [Lauraceae], 15g, CorydalisyanhusuoW.T.Wang [papaveraceae], 15g, Pharbitis nil (Linn.) Choisy [Convolvulaceae], 15g, Mirabilite, 20g, Polyporus [Polyporaceae], 30g, Alisma orientalis(Sam.)Juzep. [Alismaceae], 15g, Zingiber officinale Roscoe [Zingiberaceae], 10g, Curcuma phaeocaulis Valeton [Zingiberaceae], 10g, Toad [Bufonidae], 5 g | Y - Hospital preparations | N |
| Wen et al, 2015 | Guangdong Second Hospital of TCM | Euphorbia pekinensis Rupr. [Euphorbiaceae], 5g, Euphorbia kansui T. N. Liou ex S. B. Ho [Euphorbiaceae], 2g, Whitmania pigra Whitman [Hirudinidae], 5g | Y - Hospital preparations | N |

a: Concentration is not reported.
